# Antimicrobial activity of puffball(*Bovistella radicata*) and separation of bioactive compounds

**DOI:** 10.1186/s13568-017-0402-5

**Published:** 2017-05-19

**Authors:** Yong Ye, Kun Liu, Qinghua Zeng, Qingmei Zeng

**Affiliations:** School of Food Science and Engineering, Hefei University of Technology, 124 ShenHua Building, 193rd TunXi Road, Hefei, 230009 Anhui China

**Keywords:** Antimicrobial activity, Tinea pedis, Puffball, Fermentation supernatant, LC–MS

## Abstract

**Background:**

To test the antimicrobial activity of different extracts and fermentation broth from puffball(*Bovistella radicata*), the different extracts and fermentation broth of puffball were prepared, the active fraction was investigated by UPLC–UV–MS and semi-preparative chromatograph.

**Results:**

Through zones of inhibition (ZOI) and minimum inhibitory concentrations (MIC) tests, the supernatant of fermentation possessed best antimicrobial activity in all extracts whose MIC value is 31.2 μg/ml against *T. rubrum*, *T. mentagrophytes*, *S. aureus* and *P. aeruginosa*. And ZOI value is 29.01, 21.02, 35.02, 28.01 mm against *T. rubrum*, *T. mentagrophytes*, *S. aureus* and *P. aeruginosa*. Then we compare the puffball fermentation supernatant with blank contrast by LC–MS. There are the characteristic peaks named PBR-1 and PBR-2 with the puffball fermentation supernatant, the separation of compound PBR-1 and PBR-2 was done on semi-preparative C18 column and the MIC and ZOI of compound PBR-1 and PBR-2 are 15.6 μg/ml and 34 mm with the antifungal test.

**Conclusions:**

The fermentation supernatant and compound PBR-1 and PBR-2 have promising antifungal activity against *T. rubrum* and *T. mentagrophytes*.

## Introduction

Tinea pedis is common ailment, its treatment is a serious problem due to continuous drug resistance that microorganisms soon develop against antibiotic drugs (Subissi et al. 2010), great hope rests with traditional Chinese medicine as they contain different types of primary and secondary metabolites with antimicrobial pharmacophores (Zhang et al. 2011). Trichophyton rubrum (*T. rubrum*) and Trichophyton mentagrophytes (*T. mentagrophytes*) are main tinea pedis pathogen (Koltin and Hitchcock 1997), Candida albicans (*C. albicans*) is opportunistic fungus pathogen (Cotter and Kavanagh 2000). *S. aureus* and *P. aeruginosa* are main tinea pedis pathogen of secondary infection (Erbagci et al. 2005), Due to the emergence of resistant pathogens and the side effects of antimicrobial agents, it becomes necessary to search for new antibiotics (Cornaglia 2009). It is important to discover novel metabolites from natural resources and it is considered as an important research field.

Traditional Chinese medicine has been practiced for many centuries (Zuo et al. 2014, 2016; Xiao et al. 2016). Over the past few decades, numerous studies have been conducted on plants to explore possible candidates for antibiotic drugs. Extracts from traditional medicinal represent a continuous effort to find new compounds with the potential to act against multi-resistant microorganisms (Zampini et al. 2009). Owing to puffball diversity in biological activities and production of novel chemical compounds, it is well known that puffball’s antibacterial activity is promising (Novakovic et al. 2015).

Puffball can be found in many provinces of china. Puffball was named as *Bovistella radicata* from Shuinan town, Jiangxi province in this test. The local people use it as a remedy for anti-tinea pedis and effect is significant. According to Chinese Pharmacopoeia, puffball has been reported to have a number of functions like anti-inflammatory, stanch bleeding, cough, respiratory infections etc., however, we encountered limited published information related to antimicrobial effect of puffball, especially the antifungal activity of puffball has not been considered until now. Therefore, the present study was aimed to investigate the antimicrobial activity of puffball from Jiangxi province. In order to investigate its antimicrobial activity, main kinds of tinea pedis pathogen were selected. The antimicrobial tests were done. Isolation and identification of the activity component in puffball fermentation was done based on LC–MS. Contrast tests also demonstrated that the compound PBR-1 and PBR-2 in fermentation supernatant of puffball was active particularly against *T. rubrum.* We can make next step to indentify the structures of the compound PBR-1 and PBR-2 to develop new antibacterial substance with stronger antimicrobial activity and more beneficial characteristics.

## Materials and methods

### Chemicals and instruments

Solvents used for extraction and fractionation including ethanol, chloroform, n-butanol, ethyl acetate, aether were of AR grade. All the above reagents and potato dextrose agar (PDA), glucose (Glc) and agar were purchased from Sinopharm Chemical Reagent Beijing Co., Ltd. Standard antibiotic drugs, gentamycin and terbinafine were obtained from Pharmagen, Hefei. Laminar flow cabinet was produced by Stramline Laboratory. Petri plates and glass columns were bought from Hefei Meifeng Chemical Instrument Co. Ltd. Autoclaver was produced by Shanghai ShenAn medical equipment factory.

### Microorganisms

#### Antimicrobial microorganism (*Bovistella radicata*)

Sporophore and spore powder of puffball were obtained from pine forest of Shuinan town, Jishui county, Jiangxi province, China, with assistance of local traditional healers. After strain identification, it belongs to Agaricales order, Lycoperdaceae family, Bovistella genus, *Bovistella radicata* species, and authenticated by Professor Qingmei Zeng, College of Food Science and Engineering, Hefei University of Technology, China. Puffball(*Bovistella radicata*) specimen was dried and deposited at the herbarium of Department of Biology, Hefei University of Technology, China.

#### Test microorganisms

Test microorganisms were obtained from the Microbiology Laboratory at Department of Biology, Hefei University of Technology, Anhui. Eight strains of test microorganisms included four bacteria *Staphylococcus aureus* (ATCC 6538), *Bacillus subtilis* (ATCC 6051), *Pseudomonas aeruginosa* (ATCC 9027), *Escherichia coli* (ATCC 8739) and four fungi *Trichophyton rubrum* (ATCC 28188), *Trichophyton mentagrophytes* (ATCC 9533), *Epidermophyton floccosum* (ATCC 52066) and *Candida albicans* (ATCC 10231).

### Preparation of different extracts and fermentation from puffball

After crushed and ground, the dried sporophore of puffball was mixed with spore powder. According to established protocols (Tian et al. 2011; Zhang et al. 2012; Sen et al. 2014), each 10 mg mixed puffball powder were extracted by 200 ml ethanol/aqueous/chloroform/n-butanol/Aether/Ethyl acetate for continuously 10 h. After filtration over Whatman No. 4 paper, the extracts were concentrated under a reduced pressure at 40 °C and stored at 4 °C in sterile bottles for further use.

#### The concentrate supernatant of puffball fermentation

The mixed powder of puffball was cultured in PDB (potato dextrose broth) at 25 °C for 2 days, the fermentation was centrifuged at 7000 rpm for 20 min to get the supernatant, the supernatant was filtered over Whatman No. 4 paper, then the filtrates were evaporated on rotary evaporator at low pressure to obtain 0.5 mg/ml concentrate supernatant, the 0.5 mg/ml concentration was prepared as stock solution for further use. From stock solution, multiple proportions dilutions were prepared in water of 500, 250, 125, 62.5, 31.2, 15.6 and 7.8 μg/ml concentrations.

### Assessment of antimicrobial activity

The antimicrobial activity of puffball(*Bovistella radicata*) extracts was evaluated using two kinds of disk diffusion methods which were zones of inhibition (ZOI) (Geetha et al. 2015) and minimum inhibitory concentrations (MICs) (Negi et al. 2003), ZOI is qualitative analysis and MIC is quantitative analysis of antimicrobial activity (Dharajiya et al. 2015).

In this study, four bacterial strains and four fungal strains were used to determined the antimicrobial activity of puffball(*Bovistella radicata*). The agar cultures of *S. aureus*, *B. subtilis*, *P. aeruginosa*, *E. coli* were prepared to assess the puffball antibacterial effects. 50 ml of nutrient broth medium poured into an erlenmeyer flask, four flasks were prepared for each examined samples. The flasks including agar medium were sterilized in an autoclave at 121 °C for 15 min. For antibacterial tests, bacterial cultures were grown at 35 °C for 24 h by inoculation in Nutrient Broth (Hopper. 1992). Petri dishes with 20 ml of Nutrient Agar were prepared, previously inoculated with 100 μl of culture suspension (1%, containing 10^6^–10^7^ cfu/ml) and same volume of deionised water was used as a control. Three wells (5.0 mm in diameter) were cut from the agar at sterile condition. The prepared solutions of puffball were filled into the wells. The inoculated plates were incubated of 24 h at 35 °C, after which, it was observed for antimicrobial activity of the samples. At the end of the incubation period, the measurements were done basically from the edge of the zone to the edge of the well.

The same method was used for all the fungal samples. *T. rubrum*, *T. mentagrophytes*, *E. floccosum* and *C. albicans* were grown in Potato dextrose broth (PDB) at 25 °C for 48 h. 100 μl different fungal strain cultures (1%, containing 10^6^–10^7^ cfu/ml) were added to different flasks containing 25 ml sterile PDA at 45 °C and poured into Petri dishes. The agar was allowed to solidify at 4 °C for 1 h. The wells (5.0 mm in diameter) were cut from the agar at sterile condition. The inoculated plates were incubated of 24 h at 25 °C. PDA plates added normal saline were used as controls. The plates were then incubated at 25 °C for 2 days. The zone of inhibition (ZOI) were calculated.

For the MIC determination of a sample, agar well dilution method (Bourdeau et al. 2004) was used. Potato dextrose agar (PDA) was dissolved in distilled water with concentration 38 g/l, and autoclaved at 121 °C for 0.5 h. It was allowed to come to 50 °C before being poured into Petri plates for sample preparation. Different dilutions of each extract were prepared in this agar, with concentrations of the extract (500, 250, 125, 62.5, 31.2, 15.6 and 7.8 μg/ml). The content of each plate was mixed well and allowed to solidify. Stock solution of each extract/fraction was prepared and dilutions were made. Then, microbial cultures were transferred onto the content of each Petri plate with the help of a multipoint inoculator. Positive control and solvent control were also included. Plates were then incubated for 24 h at 37 °C, after which they were observed, the lowest concentration of antimicrobial agent at which there was no visible growth of a microorganism after incubation was taken as MIC (Petar et al. 2016) and MICs were noted.

### UPLC–UV–MS analysis of puffball

The prepared solution of fermentation was also analyzed by ultra-performance liquid chromatography (UPLC) coupled with ultraviolet (UV) and Micromass-LCT Premier time of flight (TOF) mass spectrometer (Waters, MA, USA) detector. The aim was to identify the most active fractions in the compounds of puffball. A C18 reversed-phase column (Hypersil Gold 25 mm × 2.1 mm, 1.8 μm, Thermo Scientific, Massachusetts, USA) was used with the following solvent system: A = acetonitrile, B = 0.15% ammonium acetate–water. The gradient elution was 7% A in 5 min, 7–10% A in 3 min, 10% A in 2 min, 10–15% A in 5 min, 15% A in 3 min, 15–5% A in 2 min. The LC system operated at flow rate of 1.5 ml/min for 20 min. The injection volume was 5 μl. The detection was at 330 nm. The bioactive fractions were recorded on a HPLC–MS/MS system which consisted of the HPLC Premier XE (Waters, MA, USA) connected through a split to the mass spectrometer (Waters, MA, USA) detector system operating at 45 °C. The MS analyses were performed using positive ion mode electrospray ionization mass spectrometry (ESI(+)-MS). The optimized system parameters were as follows: capillary voltage 3000 V, sample cone 30 V, source temperature 120 °C, desolvation temperature of 300 °C and collision energy 4 V, cone gas flow 70 l/h and desolvation gas flow of 350 l/h. Detection was performed in positive ion mode in the m/z range 50–1000.

#### Testify the antifungal activity of prepared compound PBR-1 and PBR-2 from puffball fermentation

The separation was carried out on semi-preparative C18 column (Phenomenex). A stock solution of supernatant of puffball fermentation was prepared. Another stock solution of blank control (PDB medium) puffball sporophore was prepared as same steps.

We compared the antifungal activity of the compound PBR-1 and PBR-2 from puffball fermentation with blank control (retention time from 8 to 12.5 min) by ZOI test, *T. rubrum* and *T. mentagrophytes* are main tinea pedis pathogens (Djeridane et al. 2006), so they were choosed as test microorganisms. *T. rubrum* and *T. mentagrophytes* were inoculated in a preliminarily melted and tempered to 45–48 °C PDA medium. The inoculated PDA media were transferred in quantity of 20 ml in sterilized Petri dishes (d = 9 cm) and allowed to solidify. After this, three wells (d = 6 mm) per dish were cut. Then the separation compound PBR-1 and PBR-2 from puffball fermentation was added into three wells (100 μl/well).

## Results

### In vitro antimicrobial assay

MIC values of prepared solution from puffball against fungi and bacteria are reported in Table 1. The most polar extracts of puffball showed antibacterial and antifungal activity against most tested microorganism. Fermentation supernatant from puffball showed good activity against the tested microorganism and the strongest activity was seen against *T. rubrum*, *T. mentagrophytes*, *S. aureus* and *P. aeruginosa* (MIC = 31.2 μg/ml), In addition, *B. subtilis* resisted all prepared solution of puffball (MIC ≥500 μg/ml). Chloroform, aether and ethyl acetate extracts of puffball demonstrated very close activities against all microorganism, all microorganism showed resistance for three extracts(MIC ≥500 μg/ml), while *T. rubrum* and *T. mentagrophytes* were also sensitive fungus to the n-butanol extract (MIC = 62.5 and 31.2 μg/ml) and aqueous extract (MIC = 31.2 μg/ml). *E. floccosum* and *C. albicans* were inhibited by high concentrations of aqueous and ethanol extracts (MIC = 125 and 62.5 μg/ml).

**Table 1:** MIC values of fermentation supernatant and extracts of puffball

Extracts/fractions of puffball	MIC (μg/ml)			
	Fungus			
	*T. rubrum*	*T. mentagrophytes*	*E. floccosum*	*C. albicans*
Fermentation supernatant	31.2	31.2	62.5	125
Ethanol extract	62.5	62.5	125	125
Chloroform extract	>500	>500	>500	>500
Aqueous extract	31.2	31.2	62.5	62.5
Ethyl acetate extract	>500	>500	>500	>500
n-butanol extract	62.5	31.2	62.5	62.5
Aether	>500	>500	>500	>500
Terbinafine	31.2	15.6	62.5	7.8

Extracts/fractions of puffball	MIC (μg/ml)			
	Bacteria			
	*B. subtilis*	*S. aureus*	*E. coli*	*P. aeruginosa*
Fermentation supernatant	500	31.2	62.5	31.2
Ethanol extract	500	62.5	125	31.2
Chloroform extract	>500	>500	>500	>500
Aqueous extract	500	62.5	62.5	15.6
Ethyl acetate extract	>500	>500	>500	>500
n-Butanol extract	500	31.2	62.5	31.2
Aether	>500	>500	>500	>500
Gentamicin sulfate	15.6	15.6	31.2	31.2

The ZOI of the fermentation supernatant from puffball exhibited strong activity against test microorganisms in Table 2. Regarding antifungal activity, the strongest antifungal activity was observed using the fermentation supernatant from puffball with the ZOI value of 29.01 ± 2.17 mm, while the strongest antibacteria activity was the ZOI value of 35.02 ± 2.03 mm. The standard drug terbinafine achieved the antifungal activity against *T. rubrum* (MIC = 31.2 μg/ml, ZOI = 26.09 ± 3.06 mm), and gentamicin sulfate achieved the highest antibacteria activity against *B. subtilis* (MIC = 15.6 μg/ml, ZOI = 43.19 ± 0.98 mm).

**Table 2:** ZOI values of fermentation supernatant and extracts of puffball

Extracts/fractions of puffball	Zone of inhibition (mm)			
	Fungus			
	*T. rubrum*	*T. mentagrophytes*	*E. floccosum*	*C. albicans*
Fermentation supernatant	29.01 ± 2.17	21.02 ± 2.05	12.03 ± 1.14	8.03 ± 1.02
Ethanol extract	10.03 ± 2.02	8.02 ± 1.12	0.00 ± 0.00	5.81 ± 0.35
Chloroform extract	0.00 ± 0.00	0.00 ± 0.00	0.00 ± 0.00	0.00 ± 0.00
Aqueous extract	15.08 ± 1.05	17.28 ± 1.67	10.02 ± 1.21	11.36 ± 1.33
Ethyl acetate extract	0.00 ± 0.00	0.00 ± 0.00	0.00 ± 0.00	0.00 ± 0.00
n-Butanol extract	18.22 ± 3.06	15.65 ± 2.16	17.12 ± 2.14	12.36 ± 1.89
Aether	0.00 ± 0.00	0.00 ± 0.00	0.00 ± 0.00	0.00 ± 0.00
Terbinafine	26.09 ± 3.06	25.04 ± 2.13	34.12 ± 3.02	38.41 ± 2.25

Extracts/fractions of puffball	Zone of inhibition (mm)			
	Bacteria			
	*B. subtilis*	*S. aureus*	*E. coli*	*P. aeruginosa*
Fermentation supernatant	8.01 ± 1.12	35.02 ± 2.03	15.03 ± 1.35	28.01 ± 1.59
Ethanol extract	0.00 ± 0.00	12.02 ± 1.12	6.03 ± 1.32	6.69 ± 1.79
Chloroform extract	0.00 ± 0.00	0.00 ± 0.00	0.00 ± 0.00	0.00 ± 0.00
Aqueous extract	12.08 ± 1.38	22.28 ± 1.12	3.02 ± 0.92	15.22 ± 1.13
Ethyl acetate extract	0.00 ± 0.00	0.00 ± 0.00	0.00 ± 0.00	0.00 ± 0.00
n-Butanol extract	0.00 ± 0.00	18.11 ± 1.02	16.32 ± 0.97	17.35 ± 1.33
Aether	0.00 ± 0.00	0.00 ± 0.00	0.00 ± 0.00	0.00 ± 0.00
Gentamicin sulfate	43.19 ± 0.98	31.03 ± 1.76	28.16 ± 2.06	32.12 ± 2.03

The date are mean ± SD of three repeats

Due to the resistance of pathogens soon developing against current drugs, it is important to questing for more effective and safer antimicrobial therapies. Natural product being reservoirs of various types of bioactive molecules are target of extensive research worldwide. In the present work, many extracts and fermentation of puffball were subjected to antimicrobial study against a number of pathogenic microorganism.

Extracts and fermentation of puffball showed antimicrobial activity in a dose-dependent manner against the test microorganisms. *T. rubrum*, *T. mentagrophytes*, *P. aeruginosa*, and *S. aureus* were the most sensitive microorganisms to the fermentation of puffball (MIC = 31.2), while the strongest activity was demonstrated against *P. aeruginosa* (MIC = 15.6 μg/ml) using the aqueous extract. Besides, fermentation and extracts of puffball showed better toxicity against *T. rubrum*, ZOI of fermentation supernatant, ethanol, aqueous and n-butanol were about 29.01, 10.03, 15.08 and 18.22 mm respectively, better antibacterial activity against *S. aureus* with ZOI of fermentation supernatant, aqueous, n-butanol and ethanol being about 35.02, 22.28, 18.11 and 12.02 mm respectively in Table 2. Notably, fermentation of puffball showed effective against bacteria and fungus. Anti-fungal and anti-bacteria compounds of puffball appeared in polar solvents and are of hydrophilic nature. This may provide a lead for an antibiotic for pathogen. In general, the fermentation supernatant and more polar extracts were better antimicrobial agents than less polar counterparts. The fermentation displayed especially notable antimicrobial efficacy, this may be due to the spore germination and the active substances are liberated by rupture of spore or the major secondary metabolites were released. *T. rubrum* and *S. aureus* were the most susceptible. Further investigation into this fraction has great prospects to yield exploitable natural products for future drugs.

### Identification of active principle using liquid chromatography–mass spectrometry (LC–MS) analysis

As stated above, based on Chinese Pharmacopoeia puffball has not been reported to have the antifungal activity. In a preliminary set of MIC and ZOI experiments, fermentation of puffball show best antifungal activity, so it is important to separate antifungal activity substance from puffball fermentation supernatant. However, in most cases, the base peak was broad with a low peak purity and the separation of these compounds with satisfactory resolution was not achieved. Besides that, anti-tinea activity maybe due to synergistic effects of different substance. The separation of purified substance is a very difficult task. Thus, we decided to validate an HPLC/UV method to determine the total content of antifungal activity and LC/MS spectra method to determine characteristic [M + H] ion m/z.

**Fig. 1:**
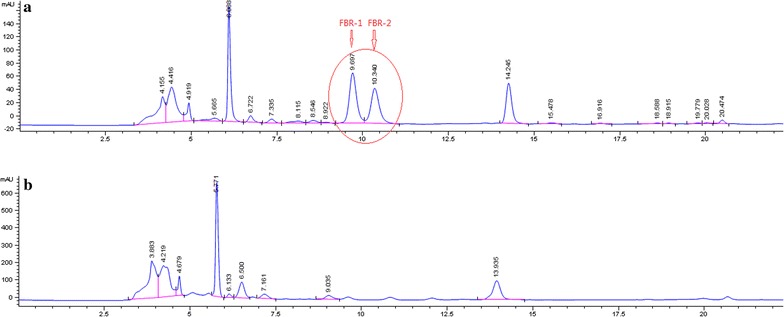
HPLC chromatograms of **a** the fermentation supernatant of puffball and **b** the blank control (PDB)

Puffball was collected in the same place in Jishui county, Jiangxi province and showed similar antifungal activities. Through contrast test, antifungal activity of puffball fermentation supernatant was done. Standard PDB medium (without puffball) was set as blank control. These supernatants were analyzed by LC–MS. Puffball and blank control presented different chromatographic profile. The chromatogram of puffball revealed the presence of two main peaks F1 (9.69 min) and F2 (10.34 min) while blank control without these peaks. The whole process is represented in the chromatograms shown in Fig. 1.

The characteristic peaks of fermentation were collected by preparation chromatographic for further studies. This compound was identified by comparison of HPLC retention time with blank control (standard PDB medium).

The difference is the characteristic peaks P1 and P2 inside the red circle between HPLC chromatograms of A and B, the component in P1 and P2 was named as PBR-1 and PBR-2, then we collected the compound PBR-1 and PBR-2, the same was for standard PDB medium (retention time from 8 to 12.5 min), antifungal activity test of the compound PBR-1 and PBR-2 was done. Test microorganisms were *T. rubrum* and *T. mentagrophytes*. Test method was ZOI (zone of inhibition). The test results are as follows.

### Antifungal activity test of characteristic peak of puffball

**Fig. 2:**
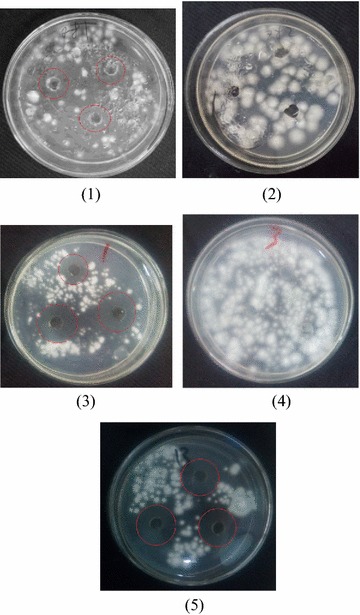
Antifungal activity of fermentation and aqueous extracts. (*1*) Contrast 1 is antifungal test of terbinafine, (*2*) contrast 2 is antifungal test of distilled water, (*3*) antifungal test of fermentation of puffball, (*4*) antifungal test of PDB medium, (retention time from 8 to 12.5 min), (*5*) antifungal activity of compound FBR-1 and FBR-2

Through ZOI test, we verified the antifungal activity of the compound PBR-1 and PBR-2 and PDB medium (retention time from 8 min to 12.5 min), the activity of antifungal was showed in Fig. 2. The red circle indicates the size of ZOI. The results showed the inhibition concentration is 50 μg/ml and the ZOI of *T. rubrum* and *T. mentagrophytes* are 34 and 27 mm respectively. The results showed in Table 3.

**Table 3:** MIC/ZOI values of compound PBR-1 and PBR-2 from puffball against pathogen

Test items		*T. rubrum*	*T. mentagrophytes*
PBR-1 and PBR-2	MIC (μg/ml)	15.6	15.6
	ZOI (mm)	34	27
Terbinafine hydrochloride	MIC (μg/ml)	31.2	15.6
	ZOI (mm)	26	25

Minimum inhibitory concentration (MIC) was carried out to determine the active concentration of the compound PBR-1 and PBR-2 that inhibited the growth of tested microorganism. MIC of the compound PBR-1 and PBR-2 against pathogenic microbes *T. Rubrum* and *T. mentagrophytes* was 15.6 μg/ml (Table 3). Notably, the compound PBR-1 and PBR-2 showed better antifungal activity than positive control (terbinafine hydrochloride) and fermentation of puffball. The compound PBR-1 and PBR-2 provide a lead result for antifungal activity.

The compound PBR-1 and PBR-2 of puffball were the most abundant antifungal activity bioactive fractions. The following molecular weight were identified based on the UV spectrum, mass spectra and fragmentation patterns.

### Purification and identification of the bioactive metabolite

**Fig. 3:**
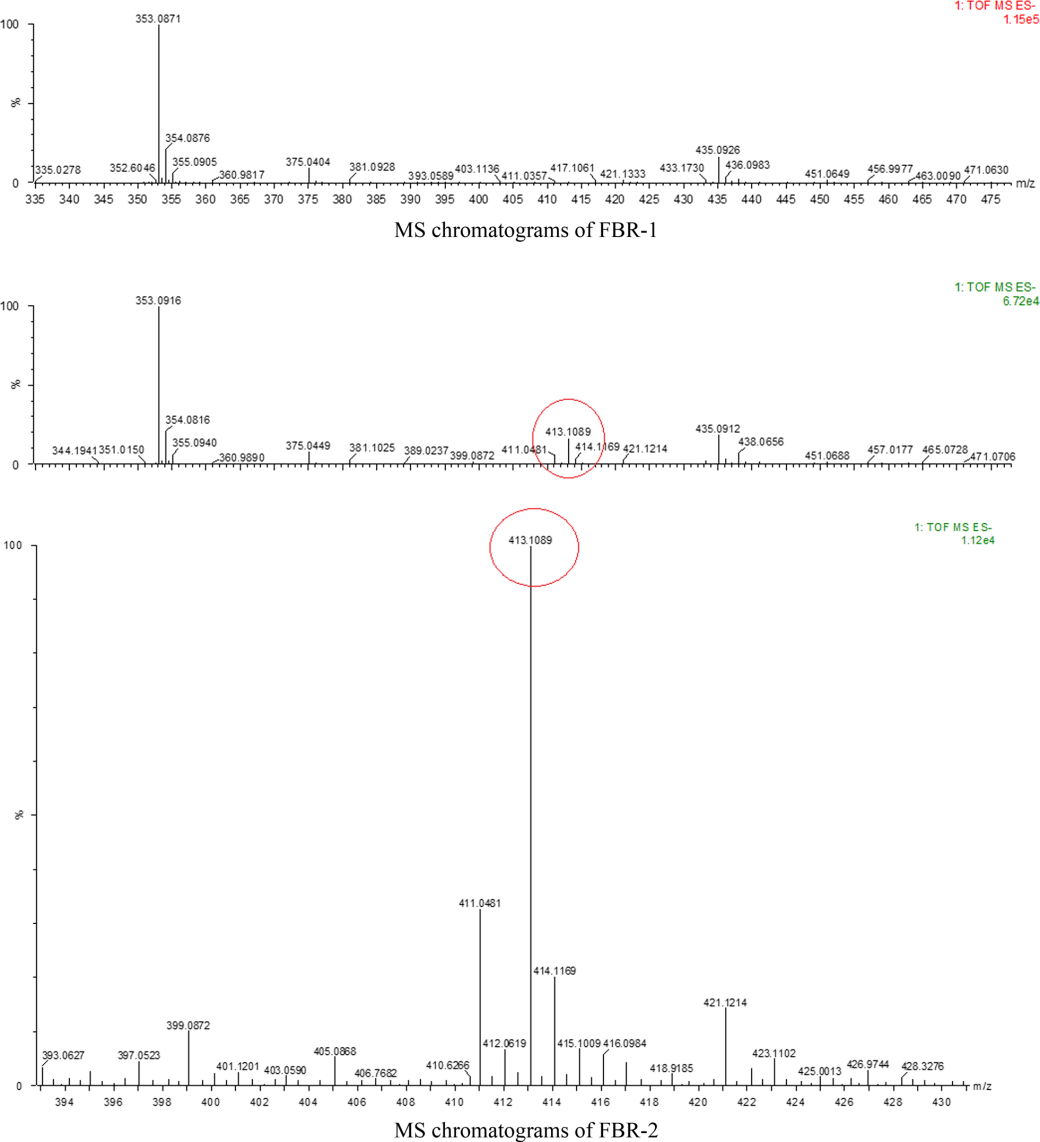
MS chromatograms of compound PBR-1 and PBR-2, the *red circle* in PBR-2 show m/z 413 (M + H) and detailed chromatograms

The compound PBR-1 and PBR-2 were subjected to LCQTOF-MS/MS analysis in positive mode ionizations. The MS of bioactive F1 in positive mode revealed a peak of m/z 353 (M + H), while F2 in positive mode revealed a peak of m/z 353 (M + H) and a peak of m/z 413 (M + H) (Fig. 3). The MS/MS of 353 resulted in formation of fragments with m/z 191, 217, 279, 299. The MS/MS of 413 resulted in formation of fragments with m/z 375, 381, 399, 411, (Fig. 3). According to mass spectra and its fragmentation pattern corresponded to bioactive fractions which have molecular mass of 353 and 413 Da (Klyba et al. 2014; Vukics et al. 2008). Machining with molecular bank, molecular formula of 353 and 413 Da are C_21_H_36_O_4_ (352.49) and C_24_H_44_O_5_ (412.58) respectively.

Fermentation supernatant of puffball has high activity against fungus, we conclude that water soluble antibacterial substances contain a lot of hydroxyl groups, based on Chinese pharmacopoeia, biological activity include steroidal, sesquiterpenes, flavonoids, alkaloids, puffball polysaccharide, fatty acid, proteins, amino acid and so on in puffball. It need to further study to identify molecular structure of antifungal properties from puffball and modify it to improve it’s anti-microbiological activity.

## Discussion

Tinea pedis fungal infection pathogens mainly include *T. rubrum*, *T. mentagrophytes* and *C. albicans*, about 70% of clinical fungal infection pathogens is T. rubrum, 15% of clinical fungal infection pathogens is *T. mentagrophytes*, *C. albicans* and other bacterials share 15%. Puffball are widespread in natural environments and puffball species have been and continue to be extensively screened for their potential for producing useful natural products (Nedelcheva et al. 2007). Previously many researchers have reported that puffball produced antibacteria activity (Canli et al. 2016; Ng et al. 2003). The present study showed that fermentation supernatant of puffball(*Bovistella radicata*) had potent antifungal and antibacterial activity with MIC value of 31.2 μg/ml, Maximum zone of inhibition (ZOI) was observed against *T. rubrum* (29.01 mm), *T. mentagrophytes* (21.02 mm), *E. floccosum* (12.03 mm), *C. albicans* (8.03 mm), *B. subtilis* (8.01 mm), *S. aureus* (35.02 mm), *E. coli* (15.03 mm), *P. aeruginosa* (28.01 mm). The antifungal activity of puffball may be attributed to an array of secondary metabolites by its spore germination, then the active substances are liberated by rupture of spore or the major secondary metabolites were produced just like steroid saponins, sesquiterpenes, flavonoids, alkaloids, puffball polysaccharide, fatty acids, amino acid, proteins and peptides and so on (Cantrell et al. 1999; Lam et al. 2001; Kamo et al. 2006). The present study revealed that puffball isolated from JiangXi province showed good antimicrobial activity against tested microbes in preliminary screening.

Steroid saponins have been reported to possess a wide range of biological activities including antibacterial (Gan et al. 2014), antifungal (Wang et al. 2015), immuno inhibitory (Nunez 1988). The bioactivity of steroidal is due to the presence of a steroid nucleus in its structure, which can be damage to the membrane and leakage of cellular materials, ultimately leading to bacteria and fungus death (Moulin-Traffort et al. 1998). So steroidal saponins maybe also have a significant role against the growth of *T. rubrum* and *T. mentagrophytes*. The role of the isolated steroidal compounds on persons’ health should be further investigated to understand its action in vivo. Sesquiterpene lactones possessed very high antifungal activity (Vajs et al. 1999). The biological activity of sesquiterpene lactones is generally attributed to the alkylating property of the α-methylene-γ-lactone moiety, and the presence of other alkylating sites (epoxides and conjugated carbonyl groups) may enhance their biological activities. Moreover, their lipophilicity seems to play an important role in antifungal activity. Since the chemical composition of the fungal cells walls is highly lipophilic, they generally represent strong barriers for the penetration of hydrophilic compounds, and the transport of polar compounds through the outer lipid layer of fungi is retarded (Skaltsa et al. 2000). Flavonoids can complex with extracellular soluble proteins and bacterial cell walls, maybe it is the reason which Flavonoids possess antimicrobial activity (Hossain et al. 2013), more lipophilic flavonoids may also disrupt microbial membranes (Ghanbari et al. 2012).

The main mechanism of the anti-microorganism of polysaccharide is to suppress signal recognition mechanism of the microorganism, which may be related with microorganism growth and mutual recognition (Schmidtchen and Malmsten 2015). Alkaloids isolated from plant are commonly found to have antimicrobial properties (Jiang et al. 2016). Berberine and hormone are important representatives of the alkaloid group. The mechanism of action of highly aromatic planar quaternary alkaloids such as berberine and harmane is attributed to their ability to intercalate with DNA (Jiang et al. 2011).

The chemical profile was analyzed in fermentation supernatant of puffball; it revealed the diversity of secondary metabolites. In future we may isolate the active molecules from the fermentation and use it as drugs for the control of microbes causing infectious diseases.

Puffball(*Bovistella radicata*) from Jiangxi province showed antibacterial and antifungal activity in contrast to the rest of puffball which had no detectable antifungal activity. Based on the Pharmacopeia, the main anti-microorganism function of puffball is anti-*S. aureus* and *P. aeruginosa*, the different results concerning the anti-microorganism activity of puffball might be due to different geographic sources of the material used, different types of strains used, and different assay methods (Awadh et al. 2003).

Since puffball demonstrated activity against the most prevalent microorganism, the use of puffball as anti-tinea pedis is validated, scientifically supported by the results obtained in this work.

## Abbreviations

UPLC: ultra-performance liquid chromatography
UV: ultraviolet
MS: mass
TOF: time of flight
ZOI: zones of inhibition
MIC: minimum inhibitory concentrations
PBR: peak of *Bovistella radicata*
PDA: potato dextrose agar
PDB: potato dextrose broth
